# Unilateral plyometric training effectively reduces lower limb asymmetry in athletes: a meta-analysis

**DOI:** 10.3389/fphys.2025.1551523

**Published:** 2025-04-09

**Authors:** Wei Sun, Hui Li, Luping Qu, Yuehui Zhou, Xiaoyang Cao, Ke Wang, Ke Li

**Affiliations:** ^1^ School of Sports Training, Tianjin University of Sport, Tianjin, China; ^2^ School of Sports Science, Qufu Normal University, Qufu, Shandong, China; ^3^ Department of Mechanical and Electrical Engineering, Changshu Riverside Vocational School, Changshu, Jiangsu, China; ^4^ Sports Teaching and Research Group, Yichang City Gezhouba Middle School, Yichang, Hubei, China

**Keywords:** lower limb asymmetry, plyometric training, complex training, athletes, meta-analysis

## Abstract

**Background:**

Lower limb asymmetry in athletes is associated with impaired performance and elevated injury risk. Plyometric training (PT) and complex training (CT) are commonly used interventions for this problem, but existing evidence on their effectiveness remains inconsistent.

**Objective:**

This meta-analysis aimed to evaluate PT and CT’s effects on athletes’ lower limb asymmetry. The findings could help optimize training protocols and reduce the risk of sports injuries.

**Methods:**

A systematic search of Web of Science, PubMed, ProQuest, Scopus, EBSCO, CNKI, and Wanfang databases was conducted up to March 2024. Two researchers independently performed the literature screening, data extraction, and quality assessment processes. A meta-analysis was conducted via Review Manager 5.3 software, including heterogeneity tests, effect size pooling, subgroup analysis, and funnel plot construction.

**Results:**

A total of eight randomized controlled trials (RCTs) involving 157 participants were included. PT effectively reduced lower limb asymmetry, particularly improving single-leg countermovement jump (SLCMJ) (SMD = 0.51, P = 0.05), single-leg broad jump (SLBJ) (SMD = 0.56, P = 0.01), and single-leg lateral jump (SLLJ) (SMD = 1.24, P = 0.01), but did not affect single-leg horizontal triple jumps (SLH3J) (SMD = 0.24, P = 0.60). In contrast, CT showed no meaningful reduction in asymmetry. Subgroup analysis indicated that unilateral PT alone significantly decreased asymmetry (SMD = 0.71, P < 0.01), whereas bilateral PT (SMD = 0.23, P = 0.45), unilateral CT (SMD = −0.15, P = 0.15) and bilateral CT (SMD = −0.09, P = 0.78) interventions all failed to demonstrate efficacy.

**Conclusion:**

Unilateral PT effectively reduces lower limb asymmetry in athletes. Coaches should integrate this method into training programs to address asymmetry-related performance deficits and injury risks. Further high-quality trials are required to validate clinical applicability.

## 1 Introduction

Lower limb asymmetry refers to the difference in performance or function of the left and right limbs (Bishop et al., 2018a; Fox et al., 2023), typically characterized by bilateral imbalances in strength, explosive power, and range of motion (Bishop et al., 2016; Moreno-Villanueva et al., 2025). Excessive asymmetry may adversely affect sports performance and increase the risk of injury (Fort-Vanmeerhaeghe et al., 2020; Helme et al., 2021; Fox et al., 2023). For example, according to Moreno-Apellaniz et al. (2024), among youth tennis players, countermovement jump (CMJ) asymmetry was significantly negatively correlated with horizontal jump performance (r = −0.47 to −0.67). In addition, when the degree of asymmetry is greater than 10%, the risk of injury to athletes increases by 4-fold (Gustavsson et al., 2006; Pardos-Mainer et al., 2020). In rehabilitation, a degree of asymmetry <10% is regarded as the reference standard for successful recovery (Kyritsis et al., 2016). Numerous studies have shown that lower limb asymmetry is prevalent among adolescents and elite athletes (Śliwowski et al., 2024; Vasileiou et al., 2024; Kiba et al., 2025), involved in various sports, such as football (Kalata et al., 2025), short track speed skating (Ojeda-Aravena et al., 2023), volleyball (Jiang et al., 2023), Basketball (Ding et al., 2024), karate (Gaweł et al., 2025). The main reasons include long-term execution of one-sided actions (e.g., basketball shooting, soccer shooting) (Hart et al., 2016; Maloney, 2019), neuromuscular fatigue (Heil et al., 2020; Fort-Vanmeerhaeghe et al., 2023) and injure (Liebermann et al., 2024; Jordan and Bishop, 2024). It is worth noting that asymmetries are exacerbated in the state of competition and fatigue (Heil et al., 2020). For example, Twible et al. (2024) found that the countermovement jump propulsive impulse asymmetry of ice hockey players significantly increased from 5.1% ± 2.6% to 6.3% ± 2.9% after a single tired on-ice training (P < 0.05).

The asymmetry index of the one-leg jump test in different directions, such as the vertical and horizontal directions, is an effective tool for quantifying lower limb performance, injury risk, and rehabilitation outcomes among athletes (Rohman et al., 2015; Guan et al., 2023; Michailidis et al., 2025). However, due to heterogeneity in athletes, sports, and testing methods (Bishop et al., 2019; D'Hondt J et al., 2024), there is no unified conclusion on the optimal threshold of lower limb asymmetry. Early studies support that a range of 10%–15% is more appropriate (Barber et al., 1990; Fousekis et al., 2012), a recent review pointed out that the evidence for universal applicability of this threshold range is still insufficient (Parkinson et al., 2021). Nevertheless, the need to improve lower limb asymmetry through training has been widely recognized (Bishop et al., 2018b; Li et al., 2021; Bettariga et al., 2022).

Plyometric training (PT) and complex training (CT) are effective methods of explosive force training; furthermore, PT and CT are important training approaches for improving asymmetry of the lower limbs among athletes because of their significant effects on improving lower limb muscle strength and high similarity with many unique technical movements (Moreno-Azze et al., 2023; Morris et al., 2022; Bettariga et al., 2023). Using the principle of the stretch-shortening cycle (SSC), PT immediately converts the elastic potential energy stored during rapid muscle elongation into kinetic energy for muscle contraction, thereby increasing the muscle strength and output power of the lower extremities (Morris et al., 2022) and enhancing the response and control of the neuromuscular system (Wang, 2012). CT refers to alternating high-intensity resistance training with reinforcement training (Hammami et al., 2019), and its theoretical basis is post-activation potentiation (PAP). This neuromuscular mechanism posits that muscle explosive power is significantly enhanced after maximum contraction (Docherty et al., 2004). This phenomenon indicates that high-intensity muscle contraction can stimulate the central nervous system, improve the force output from the higher order motor units, increase muscle contraction speed, and enhance neuromuscular strength during follow-up training (Aagaard, 2003; Docherty and Hodgson, 2007; Scott et al., 2017).

Scholars have studied the beneficial effects of PT and CT on lower limb asymmetry, but the findings are inconsistent. Sammoud et al. (2024) reported that after 8 weeks of PT intervention, long jump asymmetry was significantly reduced in preadolescent male soccer players by 4.75% (d = 0.43). Pardos-Mainer et al. (2020) found that after 8 weeks of CT intervention, there was no significant improvement in vertical and horizontal jump asymmetry in adolescent female soccer players (ES = −0.13–0.57). Zghal et al. (2019) performed a 7-week PT and CT intervention on adolescent soccer players. CT is superior to PT in improving the lower extremities’ jump height and maximum strength. With the rise of functional theory, scholars have focused on unilateral and bilateral training methods for the asymmetrical shadow of the lower limbs (Lin and Ye, 2022) and have drawn different viewpoints. Li et al. (2021) believe that unilateral training was more effective, improving lower limb asymmetry because it pertained to weak limbs and corresponded with unilateral exertion techniques in actual exercise. Bettariga et al. (2023) reported that after 6 weeks of intervention, unilateral CT significantly reduced the vertical and horizontal jump asymmetry in football players. In contrast, Ramirez-Campillo et al. (2018) conducted unilateral and bilateral CT interventions on adolescent football players for 8 weeks and found no significant difference in improving lower limb asymmetry. Overall, research on using PT and CT to improve lower limb asymmetry in athletes has progressed from general training methods to focusing on unilateral and bilateral movement patterns. However, inconsistent results meant there was a lack of clear intervention strategies. Also, few systematic reviews exist, and the specific mechanisms and differences of unilateral and bilateral PT and CT need clarification. Since meta-analysis can integrate diverse study data and quantitatively assess overall intervention effects, this study uses quantitative methods to summarize existing research findings and qualitative analysis to explore mechanisms. This approach identifies the best way to reduce lower limb asymmetry, offering evidence-based guidance to lower injury risks in athletes.

Therefore, this meta-analysis of randomized controlled trials aimed to compare the effects of unilateral and bilateral PT unilateral and bilateral CT on lower limb asymmetry in athletes. Specifically, the intervention effects of PT and CT on the asymmetry indices of the single-leg countermovement jump (SLCMJ), single-leg broad jump (SLBJ), single-leg horizontal triple jump (SLH3J), and single-leg lateral jump (SLLJ) were investigated to obtain evidence for the reasonable and scientific use of PT and CT to improve asymmetry in athletes’ lower limbs, to accurately and to reduce the risk of sports injury.

## 2 Methods

### 2.1 Bibliography retrieval

This meta-analysis was conducted in strict accordance with the PRISMA guidelines. Two independent reviewers searched the Web of Science, PubMed, ProQuest, Scopus, EBSCO CNKI, and Wanfang databases in a double-blinded manner. The databases were searched from inception to 19 March 2024. Boolean logic and truncation were used to develop the search strategies, as follows: (‘asymmetr*') AND (‘ training’ OR ‘intervention’ OR’ strength’ OR ‘jump’ OR’ change of direction’ OR ‘single leg’ OR’ unilateral’ OR ‘bilateral’ OR’ plyometric’ OR ‘complex training’) AND (‘ athletes’ OR ‘player’). To identify additional eligible studies, the reference lists of the included studies were also manually searched.

### 2.2 Inclusion and exclusion criteria

Following the PICOS principle (Population, Intervention, Comparison, Outcome, and Study Design), the inclusion criteria of the literature were formulated. The inclusion criteria were: 1) Study subjects: healthy athletes regularly participate in training and competition every week without any clinical symptoms or history of major lower limb injuries. Regardless of nationality, sex, age, sports, level and years of exercise, etc.; 2)intervention measures: The experimental group adopts at least one PT or CT as the training method. If unilateral or bilateral action mode is used, such as unilateral PT, it is also allowed to be included because it still belongs to the category of PT in essence; 3) control measures: The control group was allowed to be PT, CT, or routine training; 4) outcome index: At least one outcome index was lower limb asymmetry index before and after training, including SLCMJ, SLBJ, SLH3J, or SLLJ; and 5) study design: randomized controlled trial.

The exclusion criteria were as follows: 1) Unhealthy athletes refer to individuals with chronic diseases, acute injuries, recent surgical history, and undergoing rehabilitation training that affects standard training and lower limb function; 2) The experimental group or control group did not take PT or CT as intervention methods; 3) There is no report or insufficient data to support the calculation of lower limb asymmetry index; 4) Non-randomized controlled trials; 5) duplicate publications or studies that had inaccessible full text; and 6) reviews, conference papers and degree papers.

### 2.3 Data extraction

Two researchers independently extracted the following data from the included studies: first author, year of publication, relevant information (sports items, number of people, etc.), training methods, intervention frequency, and outcome indicators. If the lower limb symmetry index was reported in the literature, the average value was converted to an asymmetry score, and the standard deviation remained the same. In cases of missing data, the authors of the studies were contacted.

### 2.4 Evaluation of study quality

Two researchers independently assessed the risk of bias in the included studies using the Cochrane risk of bias tool. The risk of bias domains was assessed: random sequence generation, allocation concealment, blinding of participants and personnel, incomplete outcome data, selective reporting, and other biases. The risk of bias for each domain was divided into three levels: low risk of bias (meeting the low risk of five or more items), moderate risk of bias (meeting the low risk of 3-4 items), and high risk of bias (meeting the low risk of less than three items). As it is difficult to implement blinded exercise interventions, we considered the risk of bias regarding blinding to be unclear.

### 2.5 Statistical analysis

Review Manager 5.3 software was used for statistical analysis. The outcome indicators included in the literature are continuous variables assessed via different test methods. Since there are a variety of calculation formulas for limb asymmetry, such as (Nondominant limb/Dominant limb) x 100, different calculation methods may affect the results (Bishop et al., 2018c). To address this heterogeneity, we referred to a previous meta-analysis on limb asymmetry (Bettariga et al., 2022) to extract pre-intervention asymmetry values (mean ± standard deviation) reported in the original literature. To quantify the extent of the effect of the training intervention on limb asymmetry, the effect size (Hedges ‘g) was calculated as a standardized mean difference (SMD) and reported with a 95% confidence interval (95% CI). The function of SMD is to eliminate the inconsistency of calculation formulas and units between studies and make the results comparable. The specific calculation formula is:

$$g=\frac{{Mean}_{post}-{Mean}_{pre}}{{SD}_{pooled}}$$

Mean post and Mean pre represent the mean after and before the intervention, respectively, and SD pooled is the pooled standard deviation. The I^2^ test was used to assess heterogeneity among the studies; if I^2^< 50% and P > 0.1, the degree of heterogeneity was low, and the fixed effects model was adopted. The random effects model was adopted if I^2^< 50% and P ≤ 0.1. Results were visualized using a forest plot. An SMD <0.5 indicated a small effect, an SMD from 0.5–0.8 indicated a moderate effect, an SMD from 0.5–0.8 indicated a moderate effect, and an SMD ≥0.8 indicated a large effect. P ≤ 0.05 was considered to indicate statistical significance.

## 3 Results

### 3.1 Literature search results

**FIGURE 1:**
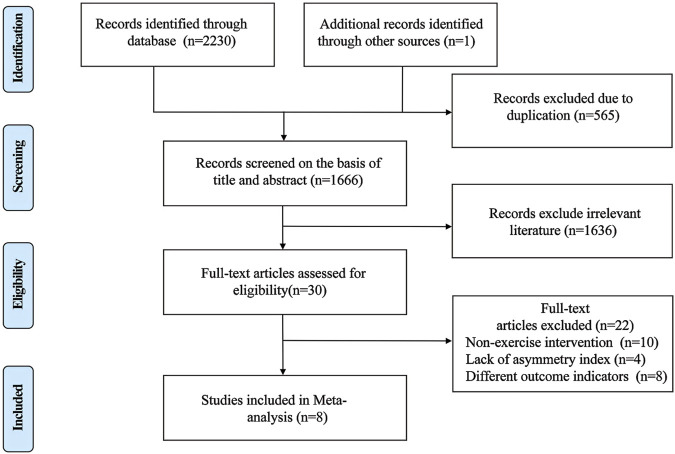
Literature screening flow chart.

The initial search yielded 2,231 relevant documents; 1,583 documents remained after removing duplicates, reviews, etc. Ultimately, eight documents were included after screening the titles, abstracts, and full texts (Figure 1).

### 3.2 Study characteristics

The majority of the eight included studies were published after 2020. The included studies involved 157 athletes, mainly individuals who participated in soccer, taekwondo, and basketball. Both PT and CT were examined in the studies, and most of the exercises were unilateral. The interventions lasted 8–10 weeks, and the intervention frequency ranged from 1–2 times per week (Table 1). Two of the included studies compared the unilateral and bilateral exercises of the same training method (Ramirez-Campillo et al., 2018; Soñén et al., 2021), and one study explored the effect of three exercise sequences of the same training method (Moreno-Azze et al., 2021). Since the current meta-analysis focused on the asymmetric changes in the same limbs before and after the intervention, the data of the above three papers were processed separately and analyzed in the following sections.

**TABLE 1:** Basic features of the included studies.

First author	Year	Sports event	Sample size	Characteristics of the training intervention			Outcome index
				Training methods	Action mode	Intervention frequency (Weeks × times)	
Sammoud	2024	Soccer	13	PT	Unlimited	8 × 2	②
Soñén	2021	Karate	22	PT	Unilateral and Bilateral	6 × 2	①②
Moreno-Azze	2023	Karate	10	PT	Unilateral	6 × 1	①②③④
Bettariga	2023	Soccer	12	CT	Unilateral	6 × 2	①②
Pardos-Mainer	2020	Soccer	19	CT	Unlimited	8 × 2	①②
Ramirez-Campillo	2018	Soccer	18	CT	Unilateral and Bilateral	8 × 2	①④
Moreno-Azze	2021	Soccer	45	CT	Unilateral	10 × 1	①②③④
Ujaković	2023	Basketball	18	CT	Unilateral	8 × 1	①

PT, plyometric training; CT, complex training; ①, single-leg countermovement jump; ②, single-leg broad jump; ③, single-leg horizontal triple jump; ④, single-leg lateral jump.

**FIGURE 2:**
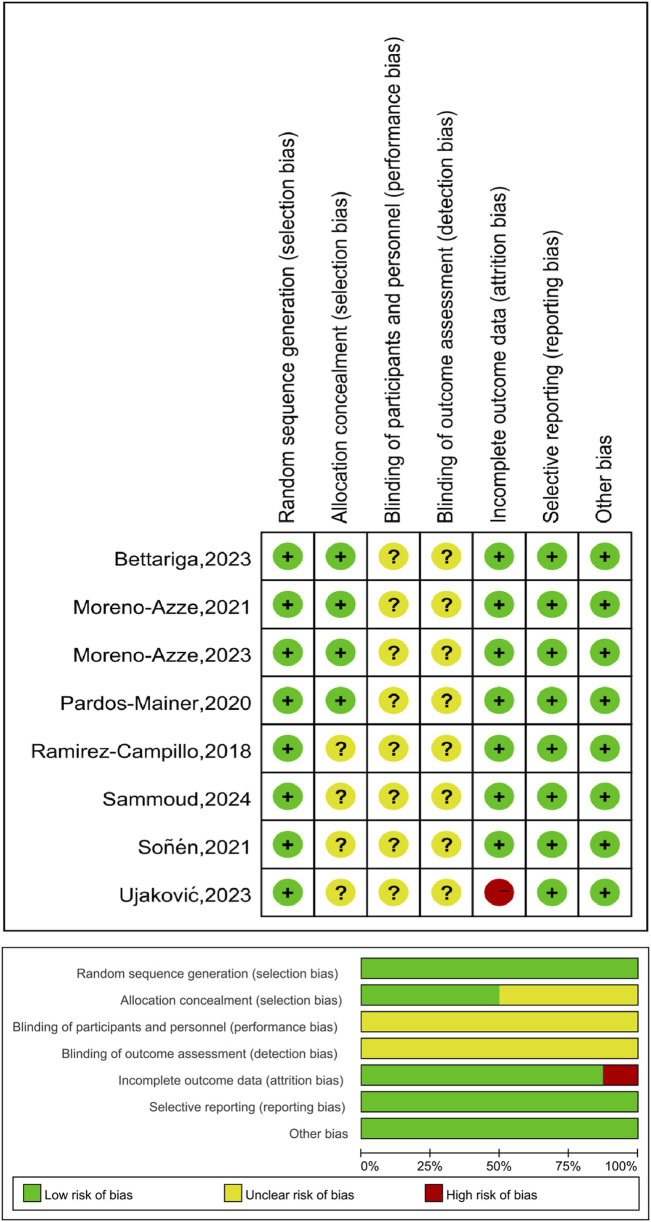
Literature quality assessment results.

Four of the included studies had a low risk of bias, the other four had a low risk of bias, and the other four had a moderate risk of bias. The results of the risk of bias assessment are shown in Figure 2.

### 3.3 Overall effect size

**FIGURE 3:**
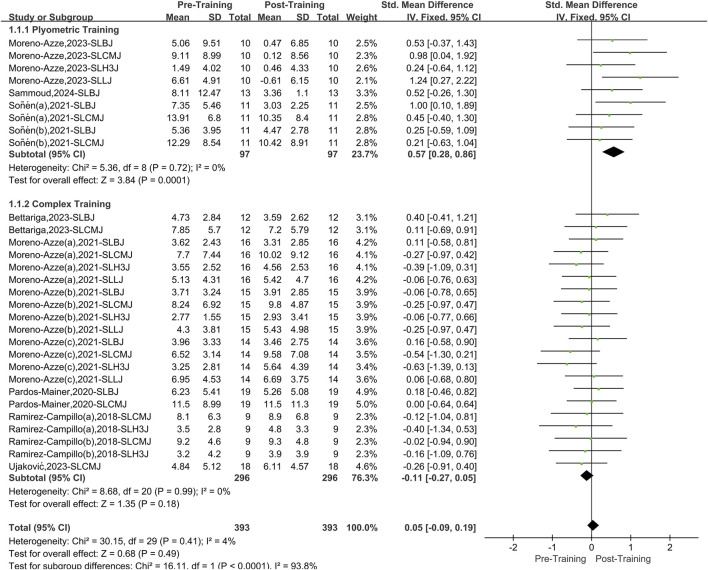
Total effect size forest picture.

A total of eight papers with 30 datasets were included, and there was a low level of heterogeneity between studies (I^2^ = 4%, P = 0.41). Therefore, a fixed effects model was used for analysis (Figure 3). When considering the overall effect of both interventions (PT and CT) on lower limb asymmetry in athletes, there was no significant effect compared to before training (SMD = 0.05, 95% CI: 0.09 to 0.19, P = 0.49). However, after stratifying the data training method, PT effectively reduces lower limb asymmetry in athletes (SMD = 0.57, 95% CI: 0.28 to 0.86, P < 0.01), yielding a medium effect size. In contrast, CT has no obvious effect on asymmetry (SMD = −0.11, 95% CI: 0.27 to 0.05, P = 0.18).

### 3.4 Meta-analysis of unilateral and bilateral plyometric training and complex training on lower limb asymmetries

#### 3.4.1 Unilateral plyometric training and unilateral complex training

**FIGURE 4:**
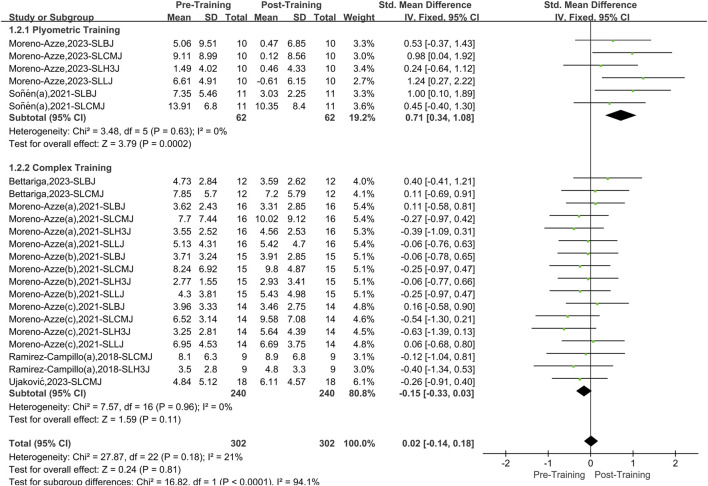
A meta-analysis of the effects of unilateral plyometric and complex training on athletes’ lower limb asymmetry.

A total of six papers with 23 datasets were included, and there was a low level of heterogeneity (I^2^ = 21%, P = 0.18). Therefore, a fixed effects model was used for analysis (Figure 4). Compared with the pretraining period, unilateral PT and CT had no significant effect on the overall intervention effect of athletes’ lower limb asymmetry. However, after stratification according to training methods, PT significantly affected lower limb asymmetry (SMD = 0.71, 95% CI: 0.34–1.08, P < 0.01), yielding a medium effect size. Unilateral CT showed no significant effect (SMD = −0.15, 95%CI: 0.33 to 0.03, P = 0.15).

#### 3.4.2 Bilateral plyometric training and bilateral complex training

**FIGURE 5:**
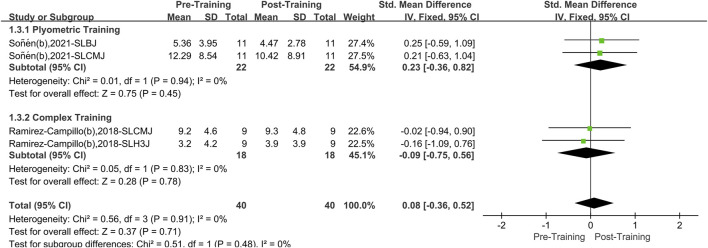
A meta-analysis of the effects of bilateral plyometric and bilateral complex training on lower limb asymmetry in athletes.

A total of two papers with four datasets were included. There was no heterogeneity (I^2^ = 0%, P = 0.91). Therefore, the fixed effects model was used for analysis (Figure 5). Compared with the pretraining period, the overall effect of bilateral PT and CT on athletes’ lower limb asymmetry was not significant. After stratification according to training method, neither bilateral PT (SMD = 0.23, 95%CI: 0.36 to 0.82, P = 0.45) nor bilateral CT (SMD = −0.09, 95%CI: 0.75 to 0.56, P = 0.78) significantly improved the effect.

### 3.5 Meta-analysis of plyometric training and complex training on different outcome indicators

#### 3.5.1 Single-leg countermovement jump

**FIGURE 6:**
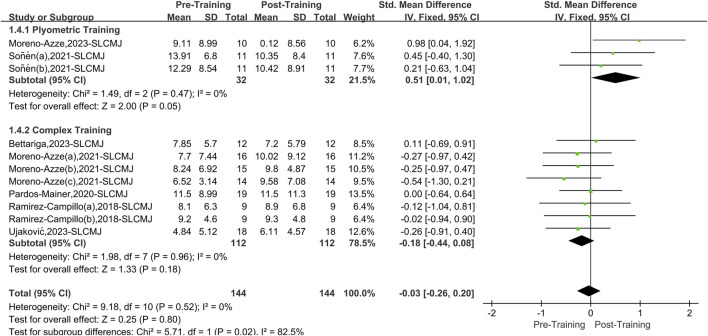
Single-leg countermovement jump forest picture.

A total of seven papers with 11 datasets examined this outcome. There was no heterogeneity (I^2^ = 0%, P = 0.52); therefore, the fixed effects model was used for analysis (Figure 6). The overall improvement in SLCMJ asymmetry between the two interventions (PT and CT) was not significant compared to pretraining (SMD = −0.03, 95% CI: 0.26 to 0.20, P = 0.80). However, after stratifying the data by training, PT effectively reduced asymmetry in athletes’ SLCMJ (SMD = 0.51, 95% CI: 0.01 to 1.02, P = 0.05), yielding a small effect size. CT did not significantly affect asymmetry in athletes’ SLCMJ (SMD = −0.18, 95% CI: 0.44 to 0.08, P = 0.18).

#### 3.5.2 Single-leg broad jump

**FIGURE 7:**
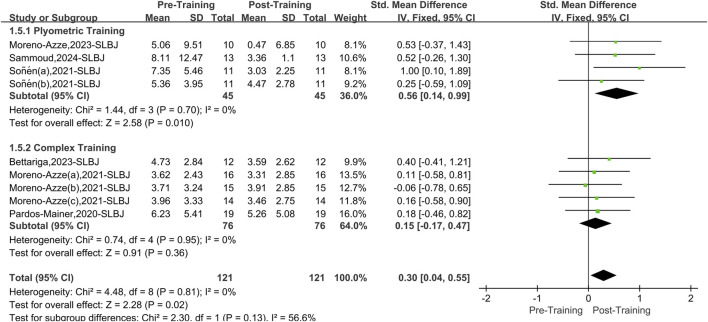
Single-leg broad jump forest picture.

A total of six papers with nine datasets examined this outcome. There was no heterogeneity among the studies (I^2^ = 0%, P = 0.72). Therefore, the fixed effects model was used for analysis (Figure 7). The two interventions (PT and CT) significantly improved overall asymmetry in the SLBJ compared to pretraining (SMD = 0.30, 95% CI: 0.04 to 0.55, P = 0.02). After stratifying the data by training, PT effectively reduced asymmetry in the SLBJ (SMD = 0.56, 95% CI: 0.14 to 0.99, P = 0.01), yielding a medium effect size. CT did not significantly affect asymmetry in the SLBJ (SMD = 0.15, 95% CI: 0.17 to 0.47, P = 0.36).

#### 3.5.3 Single-leg horizontal triple jump

**FIGURE 8:**
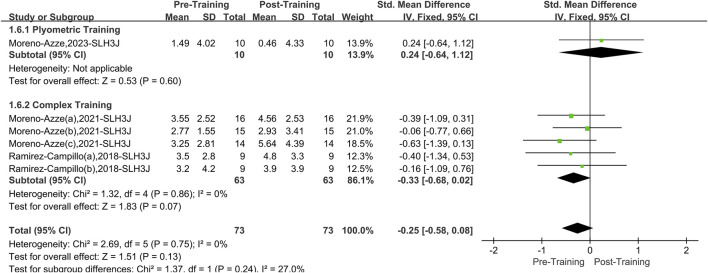
Single-leg horizontal triple jump forest picture.

A total of three papers with six datasets examined this outcome. There was no heterogeneity between the studies (I^2^ = 0%, P = 0.75). Therefore, the fixed effects model was used for analysis (Figure 8) (SMD = −0.25, 95% CI: 0.58 to 0.08, P = 0.13). The overall improvement in SLH3J asymmetry between the two interventions (PT and CT) was insignificant compared to pretraining. After stratifying the data by training method, neither PT (SMD = 0.24, 95% CI: 0.64 to 1.12, P = 0.60) nor CT (SMD = −0.33, 95%CI: 0.68 to 0.02, P = 0.07) significantly improved asymmetry in the SLH3J.

#### 3.5.4 Single-leg lateral jump

**FIGURE 9:**
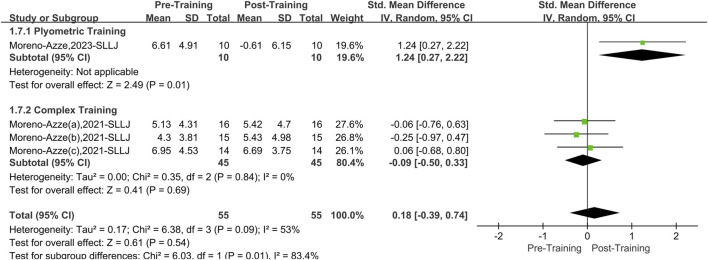
Single-leg lateral jump forest picture.

A total of two papers with four datasets examined this outcome. There was a moderate level of heterogeneity (I^2^ = 53%, P = 0.09); therefore, the random effects model was used for analysis (Figure 9). The overall improvement in SLLJ asymmetry between the two interventions (PT and CT) was insignificant compared to pretraining (SMD = 0.18, 95% CI: 0.39 to 0.74, P = 0.18). However, after stratifying the data by training, PT effectively reduced asymmetry in the SLLJ (SMD = 1.24, 95% CI: 0.272.22, P = 0.01), yielding a large effect size. CT did not significantly affect asymmetry in the SLLJ (SMD = −0.09, 95% CI: 0.50 to 0.33, P = 0.69).

### 3.6 Publication bias results

**FIGURE 10:**
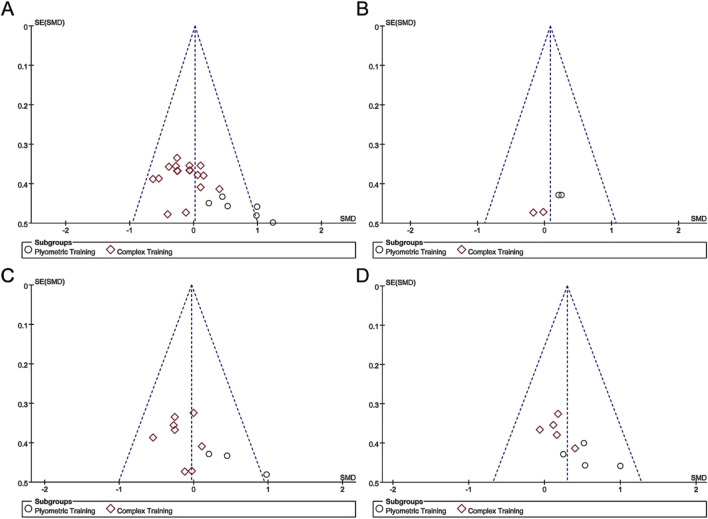
A funnel plot was constructed to examine the risk of publication bias. Figures **(A–D)** show unilateral plyometric training and unilateral complex training, bilateral rapid plyometric training and bilateral complex training, single-leg countermovement jumps, and single-leg broad jumps.

In the subgroup analysis of the included studies, there were relatively more studies examining unilateral training than bilateral training and more studies of SLCMJ and SLBJ. Therefore, a funnel plot was developed based on the abovementioned data groups. The results showed that there may be a certain degree of publication bias in the study results (Figure 10).

## 4 Discussion

This meta-analysis of eight articles found that unilateral PT significantly improved asymmetry in athletes’ lower limbs, yielding a moderate effect, whereas the effects of bilateral PT and unilateral and bilateral CT were limited. Regarding outcome indicators, PT significantly improved asymmetry in the SLCMJ, SLBJ, and SLLJ but had no significant effect on SLH3J, whereas CT did not have significant effects on the outcomes.

### 4.1 Effects of plyometric training and complex training on lower limb asymmetry in athletes

Theoretically, due to high-intensity stimulation induction and the comprehensive strength‒speed training mode, the effect of CT on asymmetry in athletes’ lower limbs should be superior to that of PT (Wang et al., 2023). However, this meta-analysis showed that PT effectively reduced athletes’ lower limb asymmetry, particularly in SLCMJ, SLBJ, and SLLJ. Our findings are consistent with recent reviews showing that PT can significantly improve athletes’ jumping performance and improve lower limb asymmetry (Bishop et al., 2018b; Bettariga et al., 2022; Xie et al., 2024). Recent randomized controlled trials have further confirmed this result (Ricart-Luna et al., 2025; Prvulović et al., 2024); for example, Prvulović et al. (2024) showed significant improvement in lower limb asymmetry in female athletes after 6 weeks of PT. In addition, the acute effect of CT on lower limb explosive force is insufficient, but the long-term effect over 10 weeks is noticeable (Hammami et al., 2017; Thapa et al., 2024) can further explain why CT has no significant improvement effect on lower limb asymmetry. For example, Hammami et al. previously published two randomized controlled trials on CT with an intervention period of 8–10 weeks, which found that CT can significantly improve the athletic performance of athletes (Hammami et al., 2022; Hammami et al., 2024). The duration of CT intervention in the literature included in this study was 6–8 weeks. This shows that providing high acceleration within the time typical for SSC is more evident than that of high-intensity stimulation provided by strength training the asymmetry in jump tests (Kons et al., 2023; Sanchez-Sanchez et al., 2024; Zhang et al., 2024).

There are several explanations to support this view. First, there is a specificity between strength training and testing (Bishop et al., 2023). The included studies were all jump tests in different directions, which depend more on high speed neuromuscular contraction ability (Kons et al., 2023). PT can ensure the activation of higher order motor units due to the high accelerations required in a short time (Norgeot and Fouré, 2024), and the conversion rate of jump performance is better (Yalçin et al., 2025). Strength training via CT can reduce the proportion of fast muscle fibers (Lamas et al., 2010). Second, the effect of PAP does not fully meet the ideal expectations. According to previous studies, the best time interval for PAP is 4–10 min (Jensen and Ebben, 2007; Jo et al., 2010), and the time range included in this study is shorter. Fatigue caused by insufficient recovery may inhibit PAP, thus mitigating CT’s effect on asymmetry in athletes’ lower limbs (Yen Yeh et al., 2024; Sousa et al., 2024). The above explanation further supports the reliability of this study’s results.

Therefore, PT intervention alone seems more effective in reducing lower limb asymmetry in athletes. This suggests that in the actual training process, coaches and athletes should consider PT an important component to reduce lower limb asymmetry and improve lower limb explosive power, significantly increasing movement speed requirements, rather than simply increasing weight.

### 4.2 Effects of unilateral and bilateral plyometric training and complex training on lower limb asymmetry in athletes

This meta-analysis revealed that unilateral PT significantly improved asymmetry in athletes’ lower limbs, whereas unilateral CT had no significant effects. Relevant studies have confirmed unilateral PT’s apparent advantages in improving athletes’ lower limb explosive power and jumping performance (Aztarain-Cardiel et al., 2025; Gharaat, 2024; Mujezinović et al., 2024). In this study, unilateral PT mainly focused on jumping, involving one-sided dominance events in karate and football (Soñén et al., 2021; Moreno-Azze et al., 2023; Sammoud et al., 2024). In these sports, explosive movements are used frequently, and perennial special training has formed a relatively stable habit of unilateral limb strength (Fox et al., 2023). The unilateral PT is highly similar to the actual force mode, which enhances athletes’ training adaptability and efficiency while improving lower limb asymmetry (Gonzalo-Skok et al., 2019; Gonzalo-Skok et al., 2017).

In contrast, the effect of low-speed strength training via unilateral CT on explosive force is limited (Kompf and Arandjelović, 2016). Additionally, considering the asymmetry changes pre and post-intervention in the included literature, unilateral PT is may more effective than unilateral CT for strengthening weak lower limb parts (Ramirez-Campillo et al., 2018; Guan et al., 2022; Moreno-Azze et al., 2023). The physiological mechanism of unilateral training, which is based on cross-transfer, means the functional improvement of the trained side positively impacts the other limb (Lee and Carroll, 2007). This shows that unilateral PT may comprehensively affect the SSC effect and cross-migration (Green and Gabriel, 2018), especially with weak lateral limb support, as athletes must jump quickly and maintain balance in unstable conditions (Drouzas et al., 2020). As body receptors receive signals and feedback from the nervous system, the brain rapidly mobilizes more motor neurons (Farthing, 2009; Manca et al., 2021), enhancing neuromuscular coordination and control (Lei and Zhang, 2018), thereby reducing sports injuries.

Bilateral PT and bilateral CT had no significant effect on improving lower limb asymmetry in athletes. This result supports previous studies, such as Cao et al. (2024), which compared 10 weeks of unilateral, bilateral, and combined PT interventions and found that only unilateral PT effectively reduced lower limb asymmetry in athletes. Gonzalo-Skok et al. (2019) did not find significant improvement in lower limb asymmetry with bilateral CT after 6 weeks of intervention, suggesting that the dominant leg may be too strong. Ottinger et al. (2023) further proved that bilateral traditional resistance training or offset loading (adding extra weight on the dominant or non-dominant side) could not effectively improve lower limb asymmetry, suggesting that unilateral training is the first choice to reduce asymmetry. This suggests that bilateral PT and CT may have two limitations in improving lower limb asymmetry: First, the action compensation phenomenon causes excessive force on the strong limb (Twible et al., 2024), resulting in insufficient stimulation of the weak limb (Qu, 2021). Second, bilateral movement induces interhemispheric activation (Qu, 2021; Shanmugam et al., 2024), simultaneous muscle contraction induces mutual inhibition between hemispheres (Anders et al., 2021), and reduced activation of high-threshold motor units (Zhang et al., 2023), bilateral force loss occurred, where the combined force of both sides is less than the sum of unilateral forces (Botton and Pinto, 2012; Rejc et al., 2024), which may weaken the improvement of lower limb asymmetry (Ottinger et al., 2023).

Therefore, in actual training, coaches and athletes should prioritize unilateral PT to improve athletes’ lower limb asymmetry and improve athletes’ explosive power and performance. Given that the asymmetry is the ratio between the two limbs (Tillin and Bishop, 2009; Qu et al., 2021) and that unilateral PT has a significant lifting effect on the weak limbs. It is recommended that the PT exercise of the weak limb be strengthened to reduce the risk of sports injury.

### 4.3 Effects of plyometric training and complex training on different outcome indicators

The study found that PT can significantly improve asymmetry in SLCMJ, SLBJ, and SLLJ but has limited effects on SLH3J asymmetry. CT did not significantly affect asymmetry in any examined jumps. Research shows that athletes’ lower limb asymmetry in the same direction is closely related to jumping performance, and interventions with similar force patterns and directions are effective (Bishop et al., 2017; Murtagh et al., 2018). The PT jump protocol included in the literature was consistent with the direction of the jump test, and the lower limb asymmetry was significantly improved; for example, Soñén et al. (2021) found that 6 weeks of depth jump training could significantly enhance SLCMJ performance and reduce asymmetry after 8 weeks of vertical and horizontal jump training. Sammoud et al. (2024) reported similar improvements in SLCMJ, SLBJ, and jump asymmetry. However, most CT programs combine multidirectional strength training with single-direction jump training, or the strength training direction differs from PT, possibly reducing the intervention effect on lower limb asymmetry (Madruga-Parera et al., 2021; Ujaković and Šarabon, 2023). Another key factor is the difference in the rate of force development RFD; higher RFD means better jump performance and asymmetry improvement (Smajla et al., 2021; Graham et al., 2024). According to the force-time curve, the shorter the ground contact time, the higher the RFD level (Scarr et al., 2021; Nagahara et al., 2025). Generally, 250 m is regarded as the critical value, and the touchdown time less than 250 m is called fast, and the reverse is called slow (Ünver et al., 2024). The PT program included in the literature in this study focuses on rapid action, such as deep jump and long jump (Sammoud et al., 2024). Enhancing RFD can improve the nerves and muscles’ rapid response ability and coordination (Scarr et al., 2021) and reduce lower limb asymmetry. However, a recent study has shown that CT has a limited effect on RFD, with little effect on overall jumping fitness (Houlton et al., 2024), and neurological fatigue caused by high-weight stimulation in the early stage of CT can also reduce RFD (Yang et al., 2025).

The SLH3J’s technical and strength demands may explain the insignificant effects of both trainings. This jump requires athletes to master rapid, continuous take-off and landing skills, strong explosive power, stability, and overcoming leg stiffness (Sharp et al., 2024; Pleša et al., 2024). Common mistakes like knee valgus during jumps (Ramzan et al., 2021) and the varied backgrounds and intervention programs of athletes in the study may have influenced the intervention’s effectiveness. Moreover, calculating lower limb asymmetry could also be a factor (Cheng et al., 2023). Post-intervention data showed a significant decrease in the average value of SLH3J asymmetry, but the standard deviation remained essentially unchanged, indicating substantial individual differences in asymmetry. Recent research highlights the y asymmetry test and measurement specificity (Bishop et al., 2021). Thus, lower limb asymmetry may be different in different directions. Using PT in the same direction can improve training efficiency and effectiveness. Since the calculation method of asymmetry may affect the evaluation results, it is recommended to adopt multiple test methods, such as jumping tests in different directions, and regularly evaluate the lower limb asymmetry from different dimensions, which is of great significance for formulating and optimizing personalized training programs and preventing sports injuries.

### 4.4 Limitations

(1) Comprehensive searches yielded limited eligible studies, particularly RCTs directly comparing the two methods, which may introduce publication bias. Furthermore, existing research lacks exploration of sport-specific or skill-level variations in lower limb asymmetry, limiting conclusion generalizability.
(2) The study strictly followed the PICOS principle but did not restrict healthy athletes’ sport, age, or gender, leading to demographic and sport-specific factors. Different kinds of literature’s varying training intensities, test methods, and formulas might skew lower limb asymmetry data results. SMD standardization reduced the impact of differences but did not eliminate all biases from tools and standards. Future research should explore these factors’ influence on lower limb asymmetry.
(3) Most studies involved 6 - to 10-week short - and medium-term interventions. Given the long-term nature of athletes’ training and competition, the effects of different training methods on lower limb asymmetry require further investigation.

### 4.5 Practical application

There are different degrees of lower limb asymmetry in athletes, and excessive differences may hurt sports performance and injury risk. Our meta-analysis shows unilateral PT effectively reduces this asymmetry, particularly in athletes who frequently use single-limb explosive power (e.g., in basketball, triple jump, and badminton) or exhibit lower limb asymmetry in jump tests (such as SLCMJ or SLBJ). Thus, when devising a targeted training plan, it is crucial to assess the direction and degree of lower limb asymmetry through multidirectional jumps and tests. Based on the assessment and the athletes’ specific conditions, weak-side unilateral PT should be strengthened to lower sports injury incidence. However, the optimal modes of unilateral PT (actions, intensity, duration) for different sports, ages, and genders are still unclear and need further study. Future research should focus on regular and dynamic assessments of lower limb asymmetry, establishing personal optimal ranges and training programs. This meta-analysis offers a theoretical basis and method for coaches and athletes to reduce lower limb asymmetry.

## 5 Conclusion

Unilateral PT effectively reduces lower limb asymmetry in athletes. Delivering high-acceleration stimuli through unstable conditions further enhances explosive power and joint stability, improving sports performance and reducing injury risks associated with unilateral overload. However, bilateral PT and unilateral/bilateral CT show no significant effects on asymmetry reduction. Given the limited number of included studies, these conclusions require verification through more high-quality randomized controlled trials.

## Data Availability

The original contributions presented in the study are included in the article/supplementary material, further inquiries can be directed to the corresponding author.
